# Argonaute Family Protein Expression in Normal Tissue and Cancer Entities

**DOI:** 10.1371/journal.pone.0161165

**Published:** 2016-08-12

**Authors:** Daniel Völler, Lisa Linck, Astrid Bruckmann, Judith Hauptmann, Rainer Deutzmann, Gunter Meister, Anja Katrin Bosserhoff

**Affiliations:** 1 Institute of Biochemistry, Emil-Fischer-Zentrum, Friedrich-Alexander-Universität Erlangen-Nürnberg, Erlangen, Germany; 2 Biochemistry Center Regensburg, University of Regensburg, Regensburg, Germany; Institut de Biologie Moleculaire et Cellulaire, FRANCE

## Abstract

The members of the Argonaute (AGO) protein family are key players in miRNA-guided gene silencing. They enable the interaction between small RNAs and their respective target mRNA(s) and support the catalytic destruction of the gene transcript or recruit additional proteins for downstream gene silencing. The human AGO family consists of four AGO proteins (AGO1-AGO4), but only AGO2 harbors nuclease activity. In this study, we characterized the expression of the four AGO proteins in cancer cell lines and normal tissues with a new mass spectrometry approach called AGO-APP (AGO Affinity Purification by Peptides). In all analyzed normal tissues, AGO1 and AGO2 were most prominent, but marked tissue-specific differences were identified. Furthermore, considerable changes during development were observed by comparing fetal and adult tissues. We also identified decreased overall AGO expression in melanoma derived cell lines compared to other tumor cell lines and normal tissues, with the largest differences in AGO2 expression. The experiments described in this study suggest that reduced amounts of AGO proteins, as key players in miRNA processing, have impact on several cellular processes. Deregulated miRNA expression has been attributed to chromosomal aberrations, promoter regulation and it is known to have a major impact on tumor development and progression. Our findings will further increase our basic understanding of the molecular basis of miRNA processing and its relevance for disease.

## Introduction

Melanoma is the most lethal form of skin cancer. Melanoma cells are derived from melanocytes; however, the exact molecular mechanisms behind tumor development are not completely understood. MiRNAs are known to play a role in this process, as evidenced by the changes in miRNA levels and target gene expression at the post-transcriptional level.

The miRNA processing cascade starts with miRNA transcription in the nucleus. The primary transcript (pri-miRNA) is further processed into the pre-miRNA hairpin intermediate. After the transfer of pre-miRNAs to the cytoplasm, mature miRNAs are formed.

Many enzymes are involved in this process and play a critical role for the miRNA production. The RNase III enzyme Drosha and its cofactor, the RNA-binding protein DiGeorge Syndrome Critical Region 8 (DGCR8; also known as Pasha in invertebrates) are responsible for processing pri-miRNAs into pre-miRNAs. Exportin 5, a nucleocytoplasmic transporter, transfers the pre-miRNA into the cytoplasm. In the cytoplasm, the RNase II enzyme Dicer processes the pre-miRNA into a double stranded miRNA:miRNA* duplex [1, 2]. Finally, one strand of the miRNA duplex is incorporated into the RISC (RNA-induced silencing complex), where it directly binds to a member of the Argonaute (AGO) protein family [3–5]. This family consists of four AGO proteins (AGO1 to AGO4) in humans, all of which are competent for gene silencing. However, only AGO2 has the ability to catalytically cleave the target gene transcript in cases of complete complementarity to the miRNA. AGO1, AGO3 and AGO4 recruit interacting proteins for downstream gene silencing events; this is also the case for AGO2 when there is only partial complementarity between the miRNA and the gene transcript. Currently, it is thought that miRNAs are randomly sorted to each AGO member without preference for a miRNA-specific AGO [6, 7].

Previous studies from our own group as well as from several other groups revealed a deregulated expression of many miRNAs in malignant melanoma cells compared to melanocytes. This observation is mainly linked to important processes that affect melanoma formation and progression (reviewed in [8–11]).

In the recent past, however, the miRNA processing proteins became a research focus being responsible for deregulation of miRNAs in cancer cells. For example, Dicer was identified up-regulated at the protein level in cutaneous melanoma [12]. It has been shown that up-regulated Dicer is associated with tumorigenesis [13, 14], although Jafarnejad et al. reported a reduction in cytoplasmic Dicer in metastatic melanoma [15, 16]. In our previous study, we showed that the observed reduction in AGO2 expression in melanoma resulted in decreased siRNA and miRNA functionality and phenotypic effects, such as a higher migratory potential of melanoma cells [17].

In this study, we further analyzed the expression and distribution of all AGO proteins in malignant melanoma cell lines and compared the expression patterns to those in other non-melanoma tumor cell lines. These insights into Argonaute expression could have consequences for miRNA-based melanoma therapies.

## Methods

### Cell culture and tissue samples

The origin and specific properties of all melanoma cell lines, NHEMs and non-melanoma cell lines used in this paper, as well as the cultivation protocol of all cells were described previously [17, 18, 19, 20]. Sample collection and handling of patient material was performed according to the ethical principles of the Declaration of Helsinki. The University of Regensburg Institutional Review Board granted approval for the project (05/077 and 10-160-0177).

### RNA isolation and reverse transcription

The isolation of total cellular RNA from all cell lines and from skin tissue samples was done with the E.Z.N.A. Total RNA Kit I (Omega Bio-Tek, VWR, Darmstadt, Germany) according to the manufacturer’s instructions. A collection of total RNA from normal tissues was derived from Clontech. Complementary DNAs were transcribed using 500 ng of total RNA via the Super Script II Reverse Transcriptase Kit (Invitrogen, Groningen, The Netherlands).

### Analysis of mRNA expression

For analysis of relative mRNA expression, quantitative real time-PCR (qRT-PCR) was performed on a LightCycler 480 (Roche, Mannheim, Germany) using 25 ng of cDNA template, 0.5 μM of forward and reverse primer and 10 μl SYBR Green Premix (Roche, Mannheim, Germany). The PCR program was performed as described in [17]. The temperatures for annealing of the respective primers (T_a_) and for detection of the PCR product (T_d_) are annotated in Table 1. Β-actin, as standard marker gene for analyses of cytoplasmic gene expression, was used for normalization. The relative mRNA expression of each gene x-fold to actin was calculated using the ΔCP method including the respective primer efficiencies (Table 1).

**Table 1:** Oligonucleotide sequences and qRT-PCR conditions.

Gene	Primer sequences (fwd/rev)	T_a_(°C)	T_d_(°C)	Eff.
Actin	1. 5’-CTA CGT CGC CCT GGA CTT CGA GC-3’ 2. 5’-GAT GGA GCC GCC GAT CCA CAC GG-3’	60	76–86	2.03
AGO1	1. 5'-TCG CCC TGC TAG CCA TCA GAC ATT-3' 2. 5'-TAC AGC GCT GCC CAG CCA CAA-3'	60	76–82	1.96
AGO2	1. 5´-GTC TCT GAA GGC CAG TTC CA-3´ 2. 5´-ATA GAG GCC TCA CGG ATG G-3´	60	76–82	1.97
AGO3	1. 5´-ATG CAA TAT GAA ACC AGC CA-3´ 2. 5´-CTG CCA AGC AAC TTG AGG TA-3´	60	76	1.93
AGO4	1. 5´-CTA GCC TGT TTC AGC CAC CTC-3´ 2. 5´-AAG GCT CAC AAC TGA CAC CC-3´	60	76–81	2.01

### Western blotting

For preparation of protein lysates, harvested cells were lysed in 200 μl RIPA buffer (Roche, Mannheim, Germany) for 15 min at 4°C with subsequent centrifugation at 13000 rpm for 10 min. For each cell line 40 μg of RIPA protein lysate (supernatant) were separated on a 10% gel via SDS-PAGE and subsequently blotted onto a PVDF membrane (Sequi-Blot^TM^ PVDF membrane and Trans-Blot^®^ Semi-Dry-system, Bio-Rad, California, USA). Unused binding sites on the membrane were blocked for 1 h with 5% non-fat dried milk solved in TBS-T. For immunodetection, the membrane was incubated with the primary antibodies anti-AGO1 (1C9, from rat, 1:50 in blocking reagent; as described in [21]), anti-AGO3 (4A11) and anti-AGO4 (6C10), both described in [22], and anti-β-actin (from mouse, 1:5000 in PBS; Sigma Aldrich, Steinheim, Germany) followed by incubation with alkaline phosphate-coupled secondary anti-mouse (1:3000 in TBS-T; Chemicon, Hofheim, Germany) or anti-rat (1:5000 in TBS-T; Sigma-Aldrich, St. Louis, MO USA) antibodies. Finally, alkaline phosphatase was used for visualization of the proteins using the substrate NBT/BCIP (Sigma Aldrich, Steinheim, Germany). The western blot bands were quantified with Image J v. 1.33 (http://rsb.info.nih.gov/ij).

### AGO-APP

AGO-APPs were conducted as described in Hauptmann et al. 2015 [23]. Briefly, cells were lysed in NET buffer (50 mM Tris pH 7.5, 150 mM NaCl, 5 mM EDTA, 0.5% NP-40, 10% glycerol, 1 mM NaF; supplemented with 0.5 mM DTT and 1 mM AEBSF). Per 50 μl Glutathione Sepharose 4 Fast Flow (GE Healthcare), a minimum of 100 μg 6×His-GST-tagged TNRC6B 599–683 was incubated with the affinity matrix for 3 h at 4°C. The beads were washed to remove excess peptide and incubated with the cell lysates for 3 h at 4°C. After washing, AGO proteins were eluted by PreScission cleavage.

### Mass spectrometry

AGO1-4 protein levels were precisely quantified by Selected Reaction Monitoring (SRM) using synthetic heavy peptides with absolute quantification as internal standards as described in Hauptmann et al. 2015 [23]. Briefly, after AGO-APP, AGO-containing protein bands were cut from the SDS gel, washed and lyophilized. For in-gel tryptic digest, 2 μg Trypsin Gold (Promega) in 100 mM NH_4_HCO_3_ was added per 100 μl gel volume. Stable-isotope labelled synthetic peptides with absolute quantification (SpikeTides TQL, JPT Innovative Peptide Solutions, Berlin, Germany) were spiked into the digests at defined amounts of 50 fmol and 100 fmol, respectively, and incubated overnight at 37°C. The Quanti-Tag of the synthetic peptides was cleaved off by trypsin releasing the following proteotypic heavy peptides: AGO1(a) NIYTVTALPIGNER, AGO1(b) VLPAPILQYGGR, AGO2(a) VLQPPSILYGGR, AGO2(b) DYQPGITFIVVQK, AGO3(a) SFFSAPEGYDHPLGGGR, AGO3(b) SANYETDPFVQEFQFK, AGO4(a) EFGIVVHNEMTELTGR, AGO4(b) QVAWPELIAIR. After collecting tryptic peptides from the in-gel digests by sequential elution two times with 50 mM NH4HCO3 and once with 50 mM NH4HCO3/50% acetonitrile, peptides were lyophilized and reconstituted in 20 μl 1% formic acid.

SRM analyses were performed on the hybrid triple quadrupole/linear ion trap mass spectrometer QTRAP4500 (Sciex) operating with the Analyst software (v. 1.6.1). In advance, paralogue-specific peptide selection and SRM-assay development had been carried out using the open source software Skyline [24] and manual evaluation. To this end an MS/MS spectral library had been created from IDA (information-dependent analysis) data of discovery runs acquired on the QTRAP4500. Proteotypic peptides with good fragmentation intensities were selected to establish specific MRM transitions including precursors with charge state 2 and 3 with 4 transitions each. The corresponding transitions of the heavy-labeled peptides were calculated by Skyline and the resulting transition list was exported to the acquisition method of the QTRAP4500 instrument software. The mass spectrometer was on-line coupled with an UltiMate 3000 RSLCnano System (Thermo Fisher) via a NanoSprayIII Ion source (Sciex). Peptides were trapped on an Acclaim PepMap100 C18 Nano Trap column (300 μm i.d. × 5 mm, Thermo Fisher) in 4% acetonitrile/0.1% formic acid. Separation of peptides was performed by reversed-phase chromatography on an analytical Acclaim PepMap C18 nano column (75 μm i.d. × 150 mm, Thermo Fisher) using a linear gradient of 4% to 40% acetonitrile in 0.1% formic acid in 45 min at a flow rate of 300 nl/min. Raw data (.wiff files) from the MRM runs were loaded into Skyline, which integrated the peak areas of the individual transitions and determined the heavy-to-light ratios of the peak areas. Further calculations as e.g. the normalization to the known amount of the spiked-in internal standard peptides were done with Microsoft Excel.

### Statistical analysis

Bar graphs, scatter plots and statistical calculations were created using the GraphPad Prism Software (GraphPad Software, Inc., San Diego, USA) and are expressed as the mean ± S.D. (range) or percent. The statistical comparison between two groups was made using the Student's unpaired t-test. One-way or two-way analyses of variance (ANOVA) were used for comparisons of more than two groups. Asterisks indicate the following p values: * = p<0.05, ** = p<0.01, *** = p<0.001, ns = not significant.

## Results

### AGO distribution in healthy tissues compared to melanocytes

**Fig 1:**
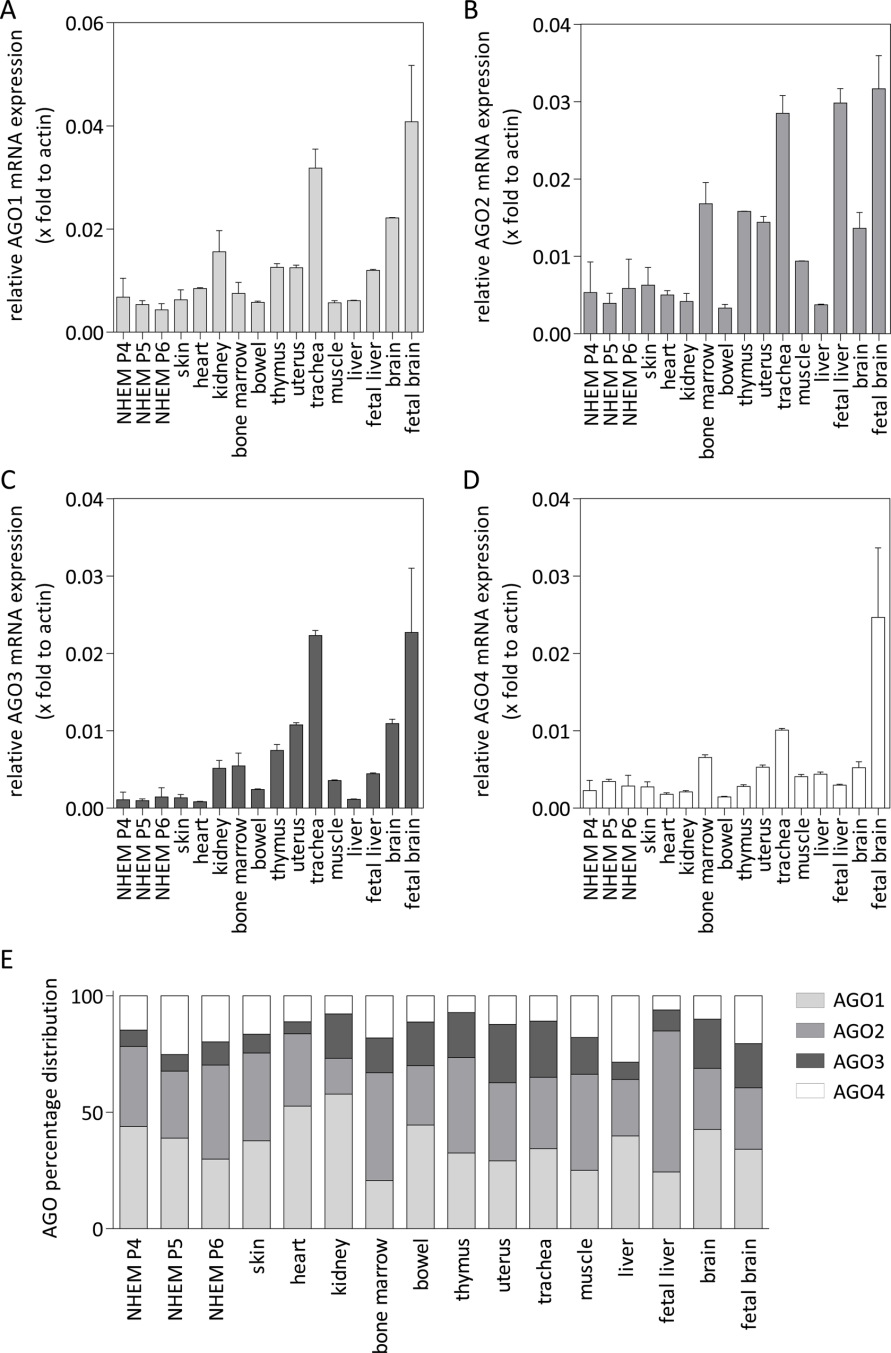
Argonaute gene expression in different human healthy tissues compared to NHEMs. Relative (A) AGO1, (B) AGO2, (C) AGO3 and (D) AGO4 mRNA expression in NHEMs (derived from different cultivation passages (P4-P6) from three different donors respectively), skin (derived from three different tissue samples) and heart, kidney, bone marrow, fetal brain, bowel, thymus, uterus, trachea, brain, muscle, bone marrow, liver, fetal liver, brain and fetal brain (derived from a total RNA bank and shown as technical replicates). (E) The compilation of percentage distribution of each AGO to the total AGO amount in the respective cell line or tissue.

MiRNAs randomly bind to all AGO proteins [7], but the effect of individual AGOs on miRNA function is poorly understood. Hauptmann et al. showed that the AGO isoform expression in a mouse organism is tissue specific and not evenly distributed [23]. This finding revealed the need to analyze the gene expression and distribution of the AGO proteins in human melanocytes (NHEMs) compared to other healthy tissues. This information is important to evaluate altered AGO expression during tumorigenesis for a specific tissue, as for melanocytes and melanoma. Therefore, we first analyzed the mRNA expression of AGO1 (Fig 1A), AGO2 (Fig 1B), AGO3 (Fig 1C) and AGO4 (Fig 1D) in different healthy tissues compared to normal human epidermal melanocytes from different donors in three different cultivation passages (NHEM P4-P6) and to skin tissue samples from different donors. We used heart, kidney, bone marrow, bowel, thymus, uterus, trachea, muscle, liver, fetal liver, brain and fetal brain derived from a total RNA bank for the AGO mRNA expression analysis. We observed relatively high mRNA expression of all the AGOs in fetal brain and trachea. AGO mRNA expression in skin, heart, bowel and muscle was comparable to melanocytes. Brain and kidney showed moderately higher AGO1 expression, fetal liver exhibited higher AGO2 expression and thymus, uterus and brain had moderately increased AGO3 expression compared to NHEMs. The AGO gene expression distribution (Fig 1E) in NHEMs was 30% to 44% AGO1, 29% to 41% AGO2, 7% to 10% AGO3 and 15% to 25% AGO4. The greatest variance was observed in AGO1 and AGO2 expression between different passages. AGO1 was the most prominent AGO in NHEM P4 and P5, skin, heart, kidney, bowel, trachea, liver, brain and fetal brain. AGO2 expression was more pronounced in NHEM P6, thymus, uterus, muscle, bone marrow and fetal liver. AGO3 expression levels in heart, skin, liver and fetal liver were comparable to NHEM and ranged between 7% and 9% whereas AGO3 expression in all other tissues varied between 15% and 25%. Fetal brain, muscle, liver, skin and bone marrow had high AGO4 expression, between 17% and 29%, whereas in all the other tissues, the AGO4 content was between 6% and 12%. Taken together, in all analyzed tissues, AGO1 and AGO2 were most prominent, but marked tissue-specific differences were identified. Furthermore, considerable changes during development were observed by comparing fetal and adult tissues (liver and brain).

### AGO distribution in melanoma and non-melanoma cancer cell lines

**Fig 2:**
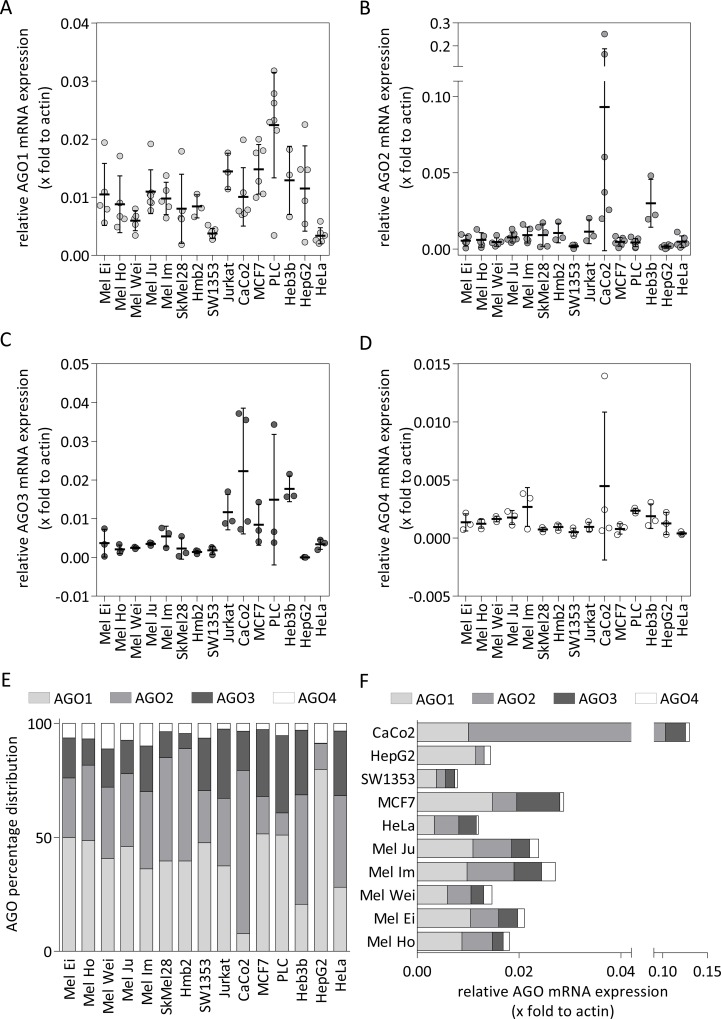
Argonaute gene expression in different melanoma and non-melanoma cell lines. Relative (A) AGO1, (B) AGO2, (C) AGO3 and (D) AGO4 mRNA expression in melanoma cell lines derived from primary tumors (Mel Ei, Mel Juso, Mel Ho and Mel Wei) and metastases (Mel Ju, Mel Im, SkMel28 and Hmb2) and other non-melanoma cell lines (HeLa, CaCo2, PLC, Jurkat, Hep3b, SW1353 and MCF7). Each point shows the measurement of one independently derived cDNA sample. Bars show mean and S.D. (E) The compilation of percentage distribution of each AGO to the aggregate AGO amount. (F) Entire mRNA expression of all four AGOs compared to actin in melanoma cell lines derived from primary tumors or metastases and in other non-melanoma cell lines.

Next, we determined the mRNA expression of AGO1 (Fig 2A), AGO2 (Fig 2B), AGO3 (Fig 2C) and AGO4 (Fig 2D) in different melanoma cell lines derived from primary tumors and metastases and in non-melanoma tumor cell lines. The relative mRNA amounts of all AGOs differed between the cell lines. The melanoma cell lines displayed AGO distributions in percent (Fig 2E) of 40% to 50% AGO1, 26% to 49% AGO2, 7% to 20% AGO3 and 4% to 11% AGO4. Mel Ei and Mel Ho cells exhibited the highest AGO1 expression (50% and 49%) whereas SkMel28 and Hmb2 showed high AGO2 expression (45% and 49%). SW1353 (human chondrosarcoma) cells also displayed AGO gene expression similar to the melanoma cell lines. CaCo2 (epithelial colorectal adenocarcinoma) cells demonstrated strong expression of AGO2 and low expression of AGO1 compared to the other cell lines. HepG2 (human hepatoma) cells showed high AGO1 mRNA expression. All other cell lines exhibited great variance stemming from the different cellular origins and donor-specific differences (see cell lines derived from hepatocellular carcinoma: PLC, Hep3B, and HepG2). Fig 2F presents the overall gene expression of all AGOs for a representative sample of the cell lines. With the exception of CaCo2, all cell lines had homogeneous overall AGO gene expression. The AGO gene expression in CaCo2 cells was increased compared to all other analyzed lines. HepG2 and HeLa cells exhibited comparable total AGO expression to Mel Wei, Mel Ei and Mel Ho cells, whereas the total AGO expression in MCF7 cells and in the melanoma cell lines Mel Ju and Mel Im was slightly increased.

### Argonaute protein expression in melanoma and non-melanoma cell lines

**Fig 3:**
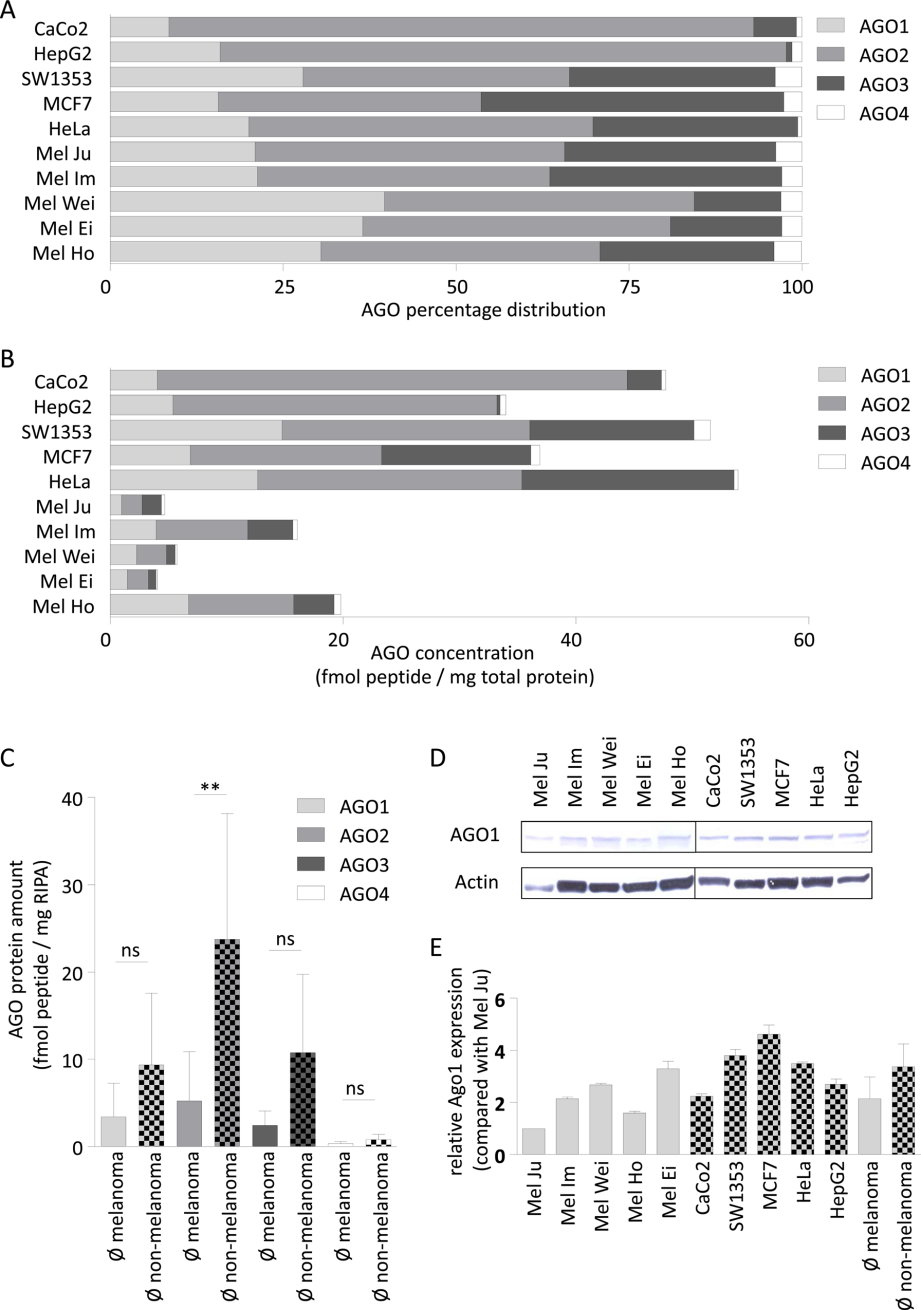
Argonaute protein distribution in melanoma and non-melanoma cell lines. (A) AGO protein percentage distribution of each AGO to total AGO protein amount in non-melanoma cell lines CaCo2, HepG2, SW1353, MCF7, HeLa and melanoma cell lines Mel Ju, Mel Im, Mel Wei, Mel Ei and Mel Ho derived from AGO-APP. (B) Total protein amount determination of each AGO enriched by AGO-APP in CaCo2, HepG2, SW1353, MCF7, HeLa, Mel Ju, Mel Im, Mel Wei, Mel Ei and Mel Ho. (C) Average AGO protein amount of each AGO in non-melanoma compared to melanoma cell lines. The reduced AGO concentration in melanoma cell lines compared to non-melanoma cell lines is only significant for AGO2. ** = p<0.01 (D) AGO1 western blot analysis and corresponding (E) Western blot quantification of melanoma cell lines (Mel Ju, Mel Im, Mel Wei, Mel Ei, Mel Ho) and non-melanoma cell lines (CaCo2, SW1353, MCF7, HeLa, HepG2). The two additional values in the AGO1 western blot quantification illustrate the average AGO1 concentration in melanoma and non-melanoma cell lines. Quantification was done relative to Actin in the respective blot.

To evaluate whether there was a correlation between the mRNA and protein data, we quantified total AGO protein expression. Based on the complexity of tumor tissue a defined analysis on protein level is not feasible in tissue. Therefore we used cell lines from specific melanoma and non-melanoma tissue to analyze the respective properties. Protein expression was analyzed by targeted mass spectrometry after AGO pull-down by a GST-tagged TNRC6B peptide that binds to all four human AGO proteins (AGO-APP) [23]. Fig 3A illustrates the relative AGO1-4 protein levels in the non-melanoma cell lines CaCo2, HepG2, SW1353, MCF7 and HeLa compared to the melanoma cell lines Mel Ju, Mel Im, Mel Wei, Mel Ei and Mel Ho.

The AGO protein expression patterns relative to each other were similar for AGO2 and AGO4 (47–55% for AGO2; 7–10% for AGO4 of the total AGO protein pool) in the melanoma cell lines. The AGO1 and AGO3 expression ratios varied slightly (26–40% for AGO1; 15–25% for AGO3). Interestingly, the two cell lines derived from metastases (Mel Im and Mel Ju) exhibited very similar AGO expression patterns.

Greater differences between the cell lines were observed at the protein level than at the mRNA level (Fig 2E). This suggests that AGO expression is regulated posttranscriptionally.

The comparison of cell lines derived from other tumors again indicated clear differences. Here, HepG2 and CaCo2 cells had the highest AGO2 protein expression (82% for HepG2; 85% for CaCo2), whereas the percentile distribution of AGO proteins in SW1353, MCF7 and HeLa cells was more similar to that in the melanoma cell lines.

Fig 3B displays the total AGO protein amount enriched by AGO-APP (in fmol protein/mg total protein) determined in each analyzed cell line. AGO-APP does not fully deplete Ago pools from the lysate in our experiments but has clearly no preference for individual Ago proteins [23]. Thus, AGO-APP-isolated proteins are quantified since this approach mirrors the cellular levels between different samples or cell lines. Interestingly, we observed in melanoma cell lines lower concentrations of all four AGO proteins (5 to 20 fmol peptide of AGO1-4 per mg total protein) than in non-melanoma cell lines. Among the non-melanoma cell lines, the lowest AGO concentration (34 fmol peptide of AGO1-4 per mg total protein) was found in HepG2 cells. HeLa cells had the highest AGO concentration, with more than 50 fmol peptide of AGO1-4 per mg total protein. There was no direct correlation between total AGO concentration and origin of the melanoma cell lines (primary tumor: Mel Ho, Mel Wei, and Mel Ei vs. metastases: Mel Im and Mel Ju).

Notably, AGO4 protein expression did not correspond with AGO4 mRNA expression. For example, the biggest disparities in protein versus gene expression were observed in the melanoma cell lines Mel Ei (-85%), Mel Wei (-87%) and Mel Im (-85%) and the non-melanoma cell lines HepG2 (-91%) and SW1353 (-78%).

Fig 3C summarizes the identified concentration of each individual AGO in the melanoma and non-melanoma cell lines. Each AGO protein was clearly down-regulated in the melanoma cell lines compared to the non-melanoma cell lines. The reduction was significant for AGO2, which exhibited the largest difference between the non-melanoma and melanoma cell lines.

To confirm the AGO mass spectrometry data, we analyzed AGO1 protein expression by western blot (Fig 3D; quantification in Fig 3E and S1B Fig). The AGO1 western blot quantification revealed a slight decrease in AGO1 expression in melanoma cell lines compared to non-melanoma cell lines, which is in accordance with the mass spectrometry data in Fig 3C. Despite this trend, absolute quantification data from mass spectrometry and western blot analyses differ (compare Fig 3B–3E). This might be due to the non-quantitative nature of the western blot validation experiments. The marked AGO2 reduction in the melanoma cell lines compared to the non-melanoma cell lines, as evidenced by AGO2 western blotting and immunofluorescence, has been previously published by our group [17]. There, an analysis of AGO2 protein level in different tissue samples derived from primary tumors or metastases showed a reduction of AGO2 compared to healthy melanocytes. AGO3 and AGO4 expression was too low for western blot analysis although highly specific and sensitive antibodies were used (S1C Fig) [18].

## Discussion

Malignant melanoma is the most aggressive form of skin cancer, and it metastasizes early and is highly resistant to current therapeutic approaches [25, 26]. Melanoma progression coincides with altered expression patterns of many miRNAs, which are also known as oncomirs [10, 27].

MiRNAs control cell proliferation, differentiation and metabolism through specific gene regulatory networks and it has been estimated that at least one-third of human protein-coding genes are miRNA targets. Furthermore, a single miRNA can target and regulate numerous genes [28, 29].

Several array studies have analyzed miRNA expression patterns in melanoma compared to melanocytes [10, 28, 30, 31]. Interestingly, it became obvious that more miRNAs are up-regulated than down-regulated in melanoma. The new finding in this study, that the total amount of AGO protein is markedly reduced in melanoma cell lines but not in other types of tumor cells, with the greatest reduction in AGO2, could serve as an explanation for the relatively up-regulated miRNA expression pattern. Due to the limitations in RISC availability, based on the reduction in AGO protein expression, only highly up-regulated miRNAs will have oncogenic functions. Even miRNAs with the same level of expression as normal melanocytes would be less effective.

The down-regulation of AGO2 in melanoma cell lines is greater than that of the other AGOs. AGO2 is the only AGO protein with cleavage activity [32]. Additional data highlight the special role of AGO2 within the AGO family. First, only the loss of AGO2 results in embryonic lethality in mice, whereas AGO1, AGO3 and AGO4 are dispensable for embryonic development [33].

Second, AGO2 processes about 27% of all endogenous pre-miRNAs to so-called ac-pre-miRNAs (AGO2-cleaved precursor miRNAs), which are intermediates in the miRNA biogenesis pathway and serve as substrates for Dicer [34]. Thereby a reduction of AGO2 results in a reduced production of Dicer matured miRNAs. This applies amongst others miRNAs to the let7 family, which have been shown to be downregulated in melanoma [35, 36].

Third, AGO2 can also process pre-miRNAs independently of Dicer, which additionally confirms the important role of AGO2 not only for miRNA based target regulation but also for miRNA biogenesis itself [37].

Typically, miRNA effects can only be observed, or are more evident, at the protein level compared to the mRNA level in melanoma. One recent example is the regulation of c-Jun by mir-125b, which can only be observed at the protein level [38]. This could also be in agreement with a strong reduction of AGO2, resulting in the use of other AGO proteins, which can only modulate translation, not direct RNA cleavage. Possible impacts of AGO2 modulation were not directly addressed in this study. Frohn and colleagues recently identified ribosomal proteins, mRNA decapping enzymes, proteins responsible for RNA binding and RISC assembly and miRNA-dependent translation regulators as direct AGO2-interacting partners independent of the miRNA loading status [39]. Potentially, these interacting partners and/or their functions could be deregulated by decreased AGO expression in melanoma.

Furthermore, it has been previously discussed whether there is a preference for a certain miRNA by a specific AGO. For example, Winter et al. reported that the miRNA let-7-3p prefers to be processed by AGO3 [40]. This study offers a differentiated expression pattern of all AGO proteins in different cell lines, tissues and also during development. This suggests a specific role for each AGO protein regarding small RNA binding and strongly argues against a random choice. Robust regulation of AGO2 could therefore result in the regulation of specific miRNAs that are dependent on AGO2. Additional research is necessary to focus on these important questions.

## Conclusion

Taken together, the results of this study reveal new insights into AGO protein expression patterns in different cell lines and tissues. The study suggests an overall reduction in total AGO protein expression in melanoma cells, with the greatest reduction in AGO2. Our findings add new information on miRNAs and miRNA processing in malignant melanoma and are important for future therapeutic applications of miRNAs in melanoma patients.

## Supporting Information

S1 Fig AGO protein expression in NHEM and melanoma cell lines. (A) Original picture of AGO1 western blot analysis shown in Fig 3D in the non-melanoma cell lines CaCo2, HepG2, SW1353, MCF7 and HeLa, the melanoma cell lines Mel Ju, Mel Im, Mel Wei, Mel Ei and Mel Ho and two NHEM samples. (B) Additional AGO1 western blot analyses corresponding to the quantification in Fig 3E. (C) AGO3 and AGO4 western blot analysis in two NHEM samples and the melanoma cell lines Mel Ju, Mel Im, Mel Ei, Mel Juso and Hmb2.
(TIF)
