# Supplementary figures and images for: Argonaute Family Protein Expression in Normal Tissue and Cancer Entities
Source: PLoS One. 2016 Aug 12;11(8):e0161165. doi: 10.1371/journal.pone.0161165 (PMC4982624; doi:10.1371/journal.pone.0161165)

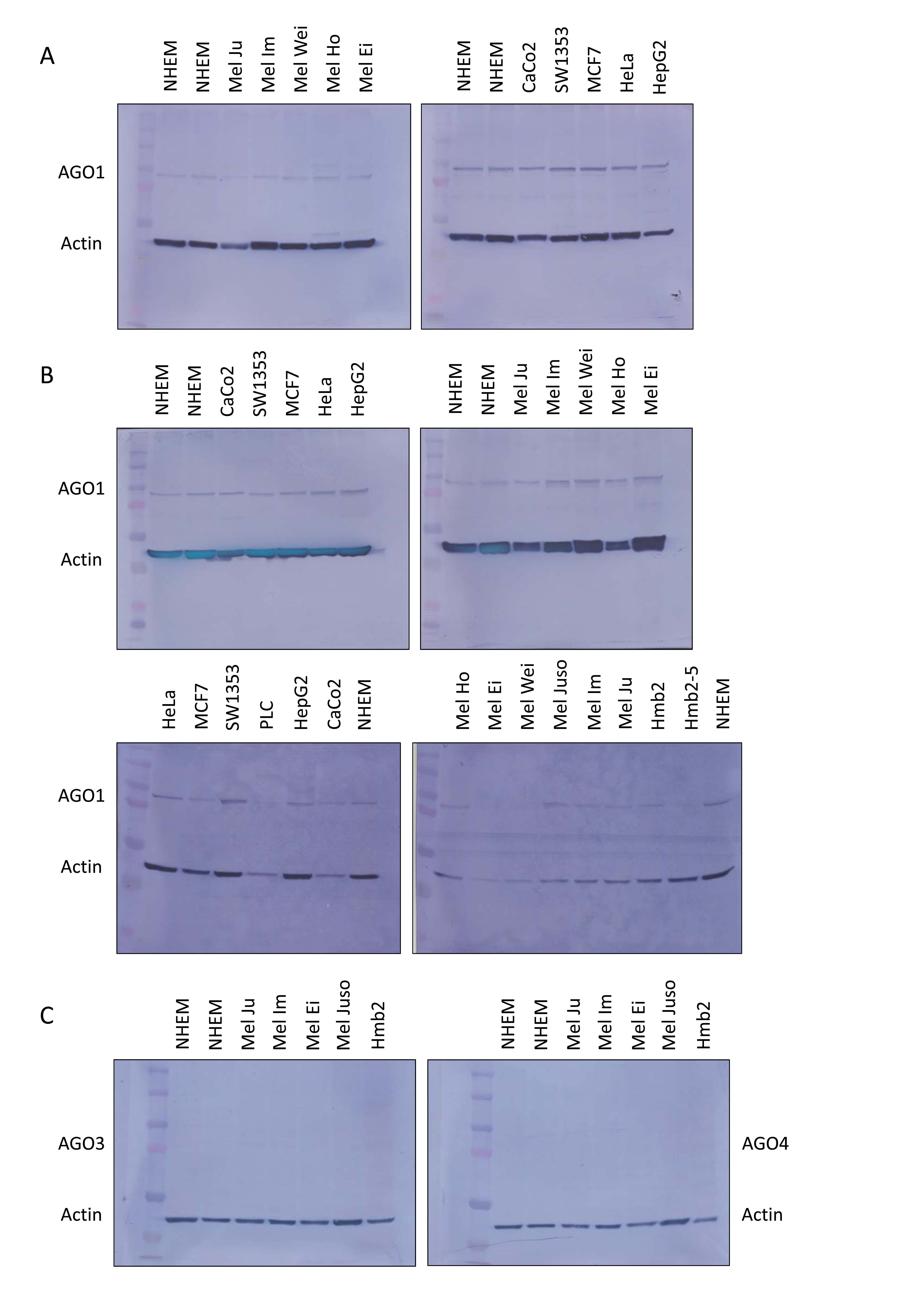

Supplement: S1 Fig — (A) Original picture of AGO1 western blot analysis shown in Fig 3D in the non-melanoma cell lines CaCo2, HepG2, SW1353, MCF7 and HeLa, the melanoma cell lines Mel Ju, Mel Im, Mel Wei, Mel Ei and Mel Ho and two NHEM samples. (B) Additional AGO1 western blot analyses corresponding to the quantification in Fig 3E. (C) AGO3 and AGO4 western blot analysis in two NHEM samples and the melanoma cell lines Mel Ju, Mel Im, Mel Ei, Mel Juso and Hmb2. (TIF) [file pone.0161165.s001.tif]
